# Economic and clinical impact of diagnostic sensitivity for the use of insertable cardiac monitors to detect atrial fibrillation in cryptogenic stroke patients in the United Kingdom

**DOI:** 10.1186/s12872-026-05871-0

**Published:** 2026-04-24

**Authors:** Klaus K. Witte, Cho-Hsuan Lee, Sarah C. Rosemas, Jonas Villinger

**Affiliations:** 1 Leeds Institute for Cardiovascular and Metabolic Medicine, University of Leeds, Leeds, UK; 2 Cardiovascular Diagnostics & Services, Medtronic International Trading Sarl, Tolochenaz, Switzerland; 3 Cardiac Rhythm Management, Medtronic, Mounds View, USA

**Keywords:** Implantable cardiac monitors (ICMs), Diagnostic sensitivity, Atrial fibrillation, Stroke, Patient outcomes, Economic evaluation

## Abstract

**Background:**

Insertable cardiac monitors (ICMs) are recommended by international clinical guidelines for prolonged cardiac monitoring in patients with cryptogenic stroke (CS). However, only LINQ ICM has been recognised as cost-effective by the National Institute for Health and Care Excellence (NICE) for this indication, as other ICMs may vary in diagnostic yield. It is unclear how this might impact patient outcomes and cost-effectiveness. This study aims to evaluate the clinical and economic impacts of different diagnostic sensitivity levels of ICMs for detecting atrial fibrillation (AF) in CS patients from the perspective of the UK National Health Service (NHS).

**Methods:**

We built a Markov model with a three-month cycle and a lifetime horizon to assess the cost-effectiveness of ICMs. Patient characteristics and detection probabilities were taken from the CRYSTAL-AF trial, which compared ICM use with the standard of care (SoC), with costs sourced from NHS. Upon detection of AF, patients transition to direct oral anticoagulants to prevent secondary ischaemic stroke (IS). Outcomes were measured in quality-adjusted life years (QALYs) and incremental cost-effectiveness ratios (ICERs). Scenario analyses were performed to examine various levels of AF detection sensitivity levels.

**Results:**

LINQ ICM versus SoC resulted in an ICER of £8,670 per QALY, below the NICE willingness-to-pay threshold. Compared with a hypothetical ICM with 30% points lower sensitivity, AF was 37.5% more likely to be detected during long-term follow-up, leading to a 9.5% reduction in the odds of recurrent IS. The lower sensitivity ICM is less cost-effective as a result, with an ICER of £13,479 per QALY versus SoC. These results remain robust under sensitivity analyses.

**Conclusion:**

Diagnostic sensitivity substantially impacts the clinical outcomes of ICMs for prolonged cardiac monitoring as well as the cost-effectiveness. ICMs with modestly lower sensitivity than LINQ are likely to be less effective at detecting AF, leading to increased stroke risks and costs, and thereby be less cost-effective.

**Supplementary Information:**

The online version contains supplementary material available at 10.1186/s12872-026-05871-0.

## Background

As a primary risk factor for stroke, atrial fibrillation contributes to cardiovascular morbidity, mortality, and healthcare costs [1–3]. Its paroxysmal and often asymptomatic nature frequently leads to under detection via conventional monitoring methods, such as 24- or 48-hour Holter monitors, thereby impeding timely stroke prevention strategies, such as oral anticoagulant therapy [4–8]. About 20–40% of ischemic strokes are cryptogenic i.e. where no clear cause is identified. Cryptogenic stroke (CS) is associated with undiagnosed AF, which is difficult to detect with short-term monitoring devices [9, 10].

Insertable cardiac monitors (ICMs) provide prolonged heart rhythm monitoring, and are increasingly used for detecting AF in patients with CS [11]. The CRYSTAL-AF trial (Clinicaltrials.gov, NCT00924638, Registration Date: 2009-06-17) demonstrated that LINQ ICMs achieved nearly ninefold greater AF detection than standard care, resulting in increased use of anticoagulants [12]. Oral anticoagulant use has been shown to significantly reduce stroke risk [8, 13]. The European Society of Cardiology (ESC) guidelines on AF diagnosis and management recommend ICMs for patients with CS, emphasising their role in identifying underlying AF [14]. Similarly, the National Institute for Health and Care Excellence (NICE) guidelines recommend the LINQ ICM for its clinical efficacy and cost-effectiveness compared with conventional ECG monitoring, whereas other ICMs are not endorsed due to insufficient evidence on diagnostic yield [15, 16]. It is likely that differences in diagnostic sensitivity alter the clinical benefit of ICMs, specifically stroke risk, and thereby the cost-effectiveness of long-term AF detection strategies.

This study aims to update the cost-effectiveness information relating to the use of Reveal LINQ to identify previously undetected AF in a population with CS and to quantify the clinical and economic impacts of differing AF detection sensitivity levels among ICMs from the perspective of the United Kingdom (UK) National Health Service (NHS).

## Methods

This study evaluated the clinical and economic implications of using ICMs with varied diagnostic sensitivity versus standard of care (SoC) for patients with cryptogenic stroke from the perspective of the UK NHS. Using Microsoft Excel, we developed a Markov model, with a lifetime horizon and 3-month cycles, consistent with the scheduled patient follow-up intervals in the CRYSTAL-AF trial and prior cost-effectiveness work of ICM in the same patient population [17]. Clinical outcomes such as life-years gained, stroke events averted and the influence of the prevention of these on costs were modelled. Key inputs were derived from the CRYSTAL-AF trial, the Oxford Vascular (OXVASC) study, and other published cost-effectiveness analyses [12, 18–20]​. In accordance with NICE guidelines, both costs and health outcomes were discounted at 3.5% annually [21, 22].

### Patient population

The model simulated a cohort of patients who had recently experienced a cryptogenic stroke or transient ischaemic attack (TIA), reflecting the population from CRYSTAL-AF [12]. Patients were divided into two groups: those receiving ICM implantation and those receiving SoC which included electrocardiograms (ECGs) and intermittent Holter monitoring (24 h to 7 days) at the discretion of their clinical team [12]. The average patient entering the model was defined based on the baseline characteristics of the CRYSTAL-AF population in the ICM group i.e. average age of 61.5, 63.5% male and CHADS_2_ score of 2.94 (based stroke risk distribution of the CRYSTAL-AF trial population) [12] (see Supplementary Table 1).

### Model structure

**Fig. 1:**
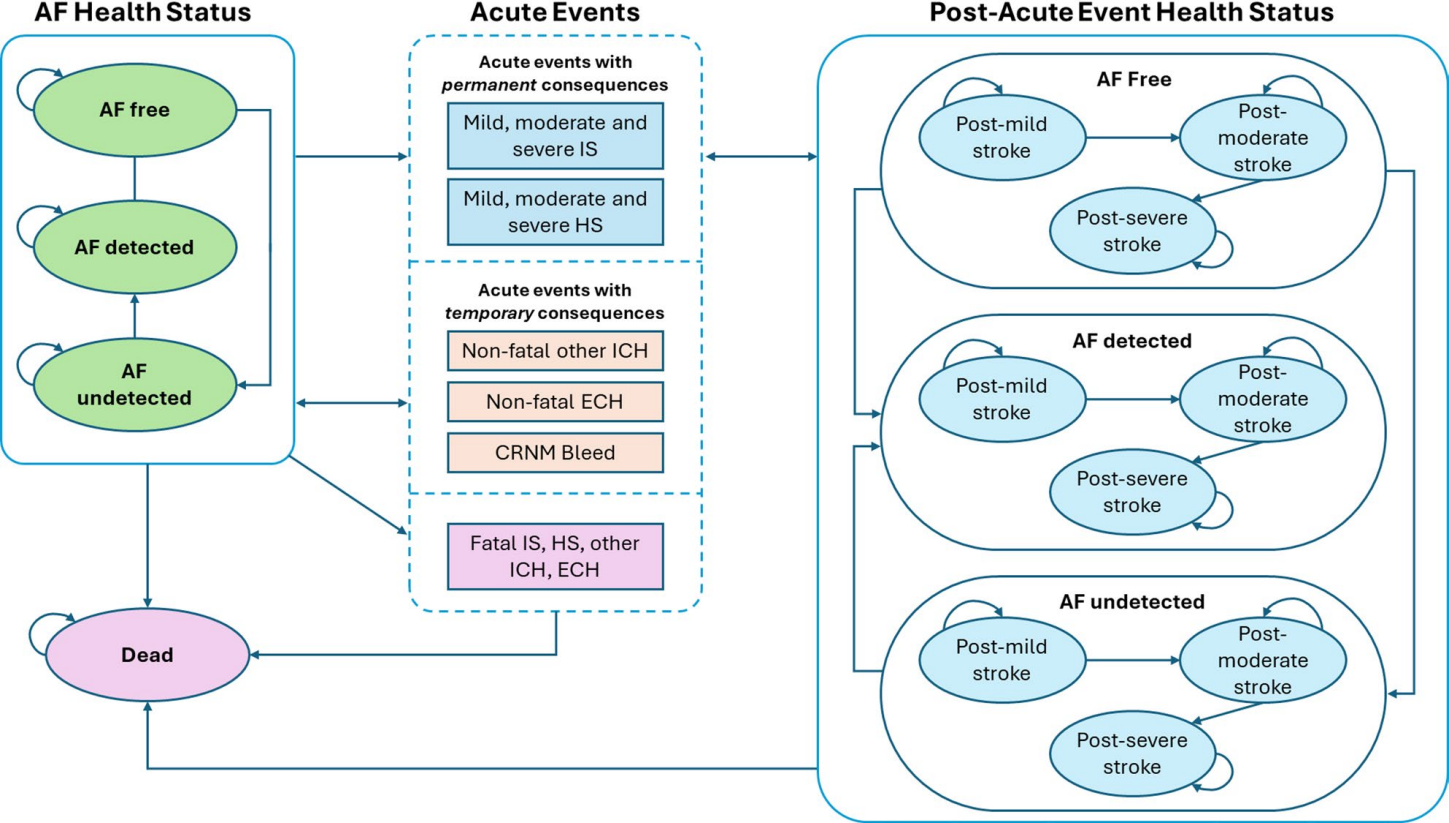
Model schematic. AF: atrial fibrillation; DOAC: direct oral anticoagulant; IS: ischaemic stroke; HS: haemorrhagic stroke; ICH: intracranial haemorrhage; ECH: extracranial haemorrhage; CRNM: clinical relevant non-major

The Markov model tracks patients across various types of health states: monitoring of AF status, acute event states, post-acute event health states, and death [17, 23] **(**Fig. 1**)**. Patients enter the model in the AF Free health state as they do not have an AF diagnosis but are beginning to be monitored with either ICM or SoC to determine if the stroke they experienced was due to underlying AF. Upon AF detection, patients move to the AF detected health state and receive oral anticoagulation (in this model DOACs) to reduce the risk of secondary stroke. Patients who have undetected AF (e.g. patients with underlying AF that has been missed by SoC, but would otherwise be detected by ICM), move to the AF undetected health state but remain on aspirin [24]. Each patient’s AF status and corresponding treatment are tracked in all health states throughout their lifetime.

Patients may also experience acute clinical events such as non-fatal bleeding, recurrent strokes, and fatal strokes. Stroke recurrence and bleeding risks were modelled on the basis of the cerebrovascular profile of the cohort as well as the risks and benefits of treatments received. In this model, patients may experience up to two recurrent strokes, each resulting in a transition to a poststroke disability state before potentially transitioning to death​.

### Model inputs

#### Incidence and detection of AF and subsequent treatment

The criteria for defining AF in this study was aligned with those used in the CRYSTAL-AF trial, which specifically refer to episodes lasting more than 30 s [12]. To estimate the relative detection yield of ICMs compared with that of conventional monitoring, the model employed a hazard ratio (HR) of 8.78 (95% CI: 3.47–22.2) derived from the CRYSTAL-AF study [12]. The detection probabilities vary between the first and subsequent cycles and differ across various CHADS_2_ subgroups [12] (Table 1). Since ICMs may not detect all AF episodes, a duration sensitivity rate of 98.9% was applied to predict the true incidence of AF [25]. The battery life for the ICMs was set at 4.5 years, extending from the previous models that used a 3-year battery life [25]. The detection probabilities beyond the trial duration are assumed to be an average of the subsequent cycles after the first three-month period at 2.4%. This is a conservative assumption close to that previously predicted [4, 17].

**Table 1:** AF detection observed between the SoC and ICM in the CRYSTAL-AF trial by the CHADS_2_ score

CHADS_2_ subgroup	Proportion of patients with AF detected by				Hazard Ratio (95% CI) - ICM vs. SoC
	SoC at 3 months	SoC at 3 years	ICM at 3 months	ICM at 3 years	
All patients (base case)*	0.9%	3.4%	8.0%	30.0%	8.78 (3.47 to 22.2)
CHADS_2_ 2	0.0%	0.0%	3.0%	18.9%	39,000,000 (0.00 to -)†
CHADS_2_ 3	1.6%	6.2%	7.8%	30.2%	4.89 (1.41 to 16.9)
CHADS_2_ 4, 5, 6	1.8%	5.4%	14.2%	45.7%	8.49 (1.97 to 36.5)

*Based on CRYSTAL-AF data

† 0 cases of atrial fibrillation were detected with the standard-of-care arm

Upon AF detection, all patients receive DOAC therapy according to NICE guidance for stroke prevention [15]. If they have experienced a haemorrhagic stroke, they generally switch back to aspirin because of the increased risk of bleeding associated with DOACs except for a proportion who resumes DOACS after 6 weeks [17]. Those without AF detected continue treatment with aspirin.

#### Ischaemic stroke risks and severity

Ischaemic strokes in the model vary in severity and are categorised as mild, moderate, severe, or fatal. The risk of ischaemic strokes is influenced by a patient’s AF status, CHADS_2_ score, use of anticoagulation treatment, and age. Various synthesised evidence was adjusted on the basis of these factors [26–30] (Supplementary Table 2). Baseline risks of ischaemic strokes for those diagnosed with AF receiving aspirin were based on a study stratified by the CHADS_2_ score [26].

The severity distribution of ischaemic stroke was considered independent of the treatment type and derived from published cost-effectiveness analyses [19, 20] (Supplementary Table 3). Although the probability of experiencing a second recurrent stroke could be higher, this model assumes that the risks of experiencing a first recurrent stroke and a second, whether haemorrhagic or ischaemic, are independent of one another. In the model, the severity distribution for the second recurrent stroke was adjusted to account for an increase in severity from the first stroke [31–33] (Supplementary Table 4).

#### Bleeding risk and subsequent treatment changes

The model categorises bleeding risk into four types, namely, intracranial haemorrhage (ICH), extracranial haemorrhage (ECH), gastrointestinal (GI) bleeding, and clinically relevant non-major (CRNM) bleeding, sourced from two cost-effectiveness analyses [19, 20]. Bleeding risks are dependent on patient age and anticoagulant treatment [27, 34–37]. The proportion of ICH that are haemorrhagic stroke and the severity of those haemorrhagic strokes, as well as the proportion of ECH patients with GI bleeding, were calculated based on the anticoagulant treatment received [19, 20] (Supplementary Table 5). Among those with other ICH and ECH, 56% and 25% respectively will permanently discontinue DOACs [38, 39]. The remaining ICH and ECH patients resume DOACs after a 6-week temporary discontinuation. Additionally, CRNM leads to temporary discontinuation of DOACs for 6 weeks, while annually, 16% of patients on DOACs are modelled to stop treatment for non-bleeding causes [20]. Those haemorrhagic stroke patients on aspirin following a bleeding event were precluded from changing to DOAC treatment even if subsequent AF detection occurred [17].

The temporary effects of non-fatal extracranial haemorrhage, other intracranial haemorrhage, and clinically relevant non-major bleeds only impact the cycle when the bleed occurs [17]. The effects of ischaemic or haemorrhagic stroke, depending on the severity of the stroke, influence costs, health-related quality of life (HRQoL), and subsequent mortality permanently [17]. The treatment effects for bleeding events and a summary of the annual risks are presented in Supplementary Tables 6 and Supplementary Table 7 [19, 20, 27, 29, 30, 35, 40–43].

#### Mortality

For mortality inputs, age-dependent background mortality rates from UK life tables were used [44]. To prevent double counting, deaths from cerebrovascular events were subtracted from the total number of deaths before estimating mortality rates, as these events are modelled separately. Data from the years 2017 to 2019 were used to avoid distortion from COVID-19-related deaths.

As this study included patients with a history of cryptogenic stroke, higher odds (HR of 1.97) for non-cerebrovascular death than in the general population were assumed [45]. After experiencing a non-fatal stroke, the risk of mortality increases depending on the treatment received as well as the severity of the stroke. The excess mortality following a recurrent non-fatal stroke is assumed as 2.71 times that of the general population [45]. These data were combined with the modified Rankin Scale (mRS) in Huybrechts’s study to adjust the HR on the basis of the severity of the stroke event, and were applied for the lifetime of the patient [46] (Supplementary Table 8).

The transition probabilities involving the detection of AF, risks of ischaemic stroke and bleeds, as well as mortality are summarised in Table 2.

**Table 2:** Key transition probabilities

Parameters	Value used	Reference – Study design
Probability of AF detection		
ICM Detected AF at month 30	0.3	[12] – RCT
HR - Base-case	8.78	[12] – RCT
HR – CHADS_2_ 2	39,000,000*	[12] – RCT
HR – CHADS_2_ 3	4.89	[12] – RCT
HR – CHADS_2_ 4+	8.49	[12] – RCT
RR – 7-day Holter vs. ICM	0.077	[47] – RCT
Risk of ischaemic stroke		
Aspirin - with AF		
CHADS_2_ 0	0.008	[26] – Pooled analysis of multiple trials
CHADS_2_ 1	0.022	[26] – Pooled analysis of multiple trials
CHADS_2_ 2	0.045	[26] – Pooled analysis of multiple trials
CHADS_2_ 3	0.086	[26] – Pooled analysis of multiple trials
CHADS_2_ 4	0.109	[26] – Pooled analysis of multiple trials
CHADS_2_ 5	0.123	[26] – Pooled analysis of multiple trials
CHADS_2_ 6	0.137	[26] – Pooled analysis of multiple trials
Treatment effects		
HR apixaban vs. aspirin	0.330	[29] – Subgroup analysis from RCT
HR apixaban vs. warfarin	0.860	[27] – Subgroup analysis from RCT
Peto^a^ OR DOAC vs. warfarin	1.030	[28, 30] – Registry study; NMA of RCTs
Risk adjustments		
HR with AF vs. without AF	1.510	[48]
Risk of bleeds		
ICH		
Annual risk - warfarin	0.012	[19, 35, 41, 42]; Weighted average across warfarin arms – CEA; RCTs
Peto OR DOAC vs. warfarin	0.470	[43]; Table 5 – NMA of RCTs
HR apixaban vs. aspirin	0.800	[43]; Table 5 – NMA of RCTs
HR apixaban vs. warfarin	0.370	[43]; Table 5 – NMA of RCTs
GI Bleed		
Annual risk - warfarin	0.011	[19, 35, 41, 42]; Weighted average across warfarin arms – CEA; RCTs
Peto OR DOAC vs. warfarin	1.220	[28,30] – Registry study; NMA of RCTs
HR apixaban vs. aspirin	0.800	[29]; Subgroup analysis from RCT
HR apixaban vs. warfarin	0.830	[27]; Subgroup analysis from RCT
CRNM Bleed		
Annual risk - warfarin	0.101	[19, 35, 41, 42]; Weighted average across warfarin arms – CEA; RCTs
Average HR DOACs vs. warfarin	0.847	[19, 35, 41, 42]; Weighted average across warfarin arms – CEA; RCTs
HR DOAC vs. aspirin	1.150	[35]; RCT
Mortality		
Bleed events		
Other ICH	0.130	[19, 20] – CEAs
Major bleed	0.020	[19, 20] – CEAs
HR versus the general population		
Post-mild stroke	2.56	[46]; Registry study
Post-moderate stroke	4.63	[46]; Registry study
Post-severe stroke	13.19	[46]; Registry study
Treatment effects		
RR aspirin vs. placebo	0.86	[43]; NMA of RCTs
RR apixaban vs. aspirin	0.85	[43]; NMA of RCTs
RR apixaban vs. warfarin	0.89	[43]; NMA of RCTs
RR DOAC vs. warfarin	0.91	[43]; NMA of RCTs

^a^Peto’s method uses an inverse variance approach and utilizes an approximate method of estimating the log odds ratio considering different weights to pool odds ratios

* 0 cases of atrial fibrillation were detected with the standard-of-care arm

*AF* atrial fibrillation, *CEA* cost-effectiveness analysis, *CRNM* clinically relevant non-major, *DOAC* direct anticoagulant, *GI* gastrointestinal, *HR* hazard ratio, *ICH* intra-cranial haemorrhage, *NMA* network meta-analysis, *OR* odds ratio, *RR* relative risk, *RCT* randomized controlled trial

#### Health state utilities

To estimate QALYs, the model applies baseline EQ-5D data from the CRYSTAL-AF trial [12, 49]. HRQoL was defined on a scale from 0 (equivalent to death) to 1 (perfect health). Evidence for acute stroke events and poststroke utility from the OXVASC study was used to obtain HRQoL values. The OXVASC study is an ongoing UK population-based cohort study of assessing the incidence, causes and long-term outcomes of acute vascular events in a population of over 91,000 patients in Oxfordshire County. Given the studied population, it was closer to the UK perspective than other studies [50]. Disutility values associated with bleeding events were drawn from other published models (Supplementary Table 9) [12, 19, 49, 50]. Since age and sex interact with patients’ utilities and those reported in the OXVASC are based on a population with a mean age of 75, these utility values were adjusted with established formulae to reflect the characteristics of this cohort [18, 49, 50]. The derived utility multipliers are available in Supplementary Table 10 [49, 51].

The temporary disutility caused by ischaemic or haemorrhagic stroke and other intracranial haemorrhages was expected to last for one cycle [17, 23, 52], after which the poststroke utilities are applied. Conversely, it was assumed that the disutility for extracranial haemorrhage would last for two weeks, and for clinically relevant non-major bleeds, it would last for two days. These assumptions aligned with those from earlier work [17, 23, 52]. 

#### Resource use and costs

##### Standard-of-Care (SoC) arm

In the SoC arm, the cardiac monitoring included infrequent use of ECGs and 24-hour, 48-hour, and 7-day Holter monitoring of varying durations, with the frequency of tests drawn from the CRYSTAL-AF trial [12]. The tests were separated into first, second, and third years, with the third year reflecting long-term follow-up (Supplementary Table 11) [17]. The unit cost for all monitoring tests was based on the Healthcare Resource Groups (HRGs) code EY51Z [53, 54].

##### LINQ ICM arm

For the ICM arm, the costs included one-time acquisition, insertion, and removal of the ICM, with monitoring as an additional cost. As observed in the trial, an annual 2.4% risk of unplanned removal was assumed, covering clinical, technical, and personal reasons[12]. Monitoring was assumed to occur biannually, with costs comprising a mix of consultant-led (46%) and non-consultant-led (54%) sessions, based on previous literature of clinical practice in the UK [55]. After the removal of the ICM following the end of its battery life, all patients, whether they had been diagnosed with AF or not, ceased to be monitored with an ICM.

##### Drug and INR monitoring costs

Upon detection of AF, patients switched to DOAC treatment, which included an average of all licensed agents: dabigatran, rivaroxaban, apixaban, and edoxaban [17]. Use of warfarin instead of DOAC was also tested in the sensitivity analyses. The administration frequency and costs of drugs were drawn from the British National Formulary [56–61]. According to clinical requirements [62] and NHS activity levels [19, 54], patients treated with warfarin were assumed to require 18 international normalised ratio (INR) monitoring visits. The intervention, treatment, and monitoring costs are summarised in Table 3.

**Table 3:** Cost of interventions and treatment

Cost item	Mean cost (£)	Source
Device related		
ICM acquisition and implantation	£1,769	[53]; HRG EY12A & EY12B day case weighted average
ICM removal	£868	[53]; HRG EY13Z total averaged
Monitoring and follow-up consultation		
Cost per interrogation	£80	[53]; HRG WF01 Outpatient attendances 320 Cardiology Service
Unit cost of ECG/Holter monitoring	£177	[53]; HRG EY51Z
INR monitoring (per cycle)	£81	[19]
Drug cost		
Aspirin 75 mg	£2.1	[60]
Warfarin 5 mg	£5.5	[61]
Averaged DOAC cost*	£161.1	[56–59]

*ECG* electrogram, *INR* international normalized ratio, *DOACs* direct oral anticoagulants

* Average cost of dabigatran, rivaroxaban, apixaban and edoxaban

##### Stroke and bleed event costs

Acute stroke events incurred immediate costs during the cycle, and if long-term health impacts occurred, relevant costs were accrued to the “poststroke” health state. These costs were based on the OXVASC study and adjusted for inflation to the year 2023 [50, 54].

Bleeding event costs were calculated as weighted averages of several HRG codes and settings from the latest NHS Reference Costs database, aligning with the assumptions from previously published studies [23, 52, 53]. All the stroke and bleeding event costs, as well as the utility values, are summarised in Table 4.

**Table 4:** Costs and utilities of health states and events

Event cost and mean utility	Mean cost (£)	Mean utility/ disutility	Standard error	Source
Baseline utility	-	0.774	0.0134	[12]
Disutility of AF detected	-	-0.014	0.0192	[18, 50]
Stroke events				
Mild IS	£4,652	0.730	0.0140	[18, 50]
Moderate IS	£24,271	0.500	0.0370	[18, 50]
Severe IS	£33,150	0.130	0.0570	[18,50]
Fatal IS	£4,185	0	-	[18, 50]
Disutility for all recurrent acute stroke events	-	-0.150	0.0395	[18, 50]
Poststroke events (IS or HS)				
Mild IS	£730	0.727	0.0122	[18, 50]
Moderate IS	£1,424	0.582	0.0355	[18, 50]
Severe IS	£2,163	0.397	0.0655	[18, 50]
Disutility for all recurrent stroke events – post-acute period	-	-0.068	0.0242	[18, 50]
Bleed events				
Mild HS	£13,547	0.730	0.0140	[18, 50]
Moderate HS	£34,802	0.500	0.0370	[18, 50]
Severe HS	£58,869	0.130	0.0570	[18, 50]
Fatal HS	£2,178	0	-	[18, 50]
Other ICH	£4,799*	0.700	0.0930	[18, 50, 53]
Other ECH	£6,160**	-0.151	0.0401	[18, 50, 53]
GI bleed	£2,333***	-0.151	0.0401	[18, 50, 53]
CRNM bleed	£691†	-0.058	0.0173	[18, 50, 53]

*AF* atrial fibrillation, *IS* ischaemic stroke, *HRG* healthcare resource group, *HS* haemorrhagic stroke, *GI* gastrointestinal, *CRNM* clinically relevant non-major

*weighted average of HRG codes AA23C–G; **weighted average of HRG codes BZ24D–G, EB14A–E, FF51A–J, HC28H–M and HD24D–H; ***weighted average of HRG codes FD03A–H

†weighted average of HRG codes FD03A–H, CA23Z and LB38C–G

### Analyses

The base-case analysis evaluated the cost-effectiveness of LINQ ICM compared with SoC over the patient’s lifetime from the UK NHS perspective in the CRYSTAL-AF population. We estimated the incremental cost-effectiveness ratio (ICER), which synthesises QALYs and healthcare costs, and used payers’ willingness-to-pay thresholds of £20,000 and £30,000 per added QALY.

#### Sensitivity analyses

Deterministic and probabilistic sensitivity analyses were performed to test the robustness of the findings. A deterministic one-way sensitivity analysis (OWSA) was conducted on all the input parameters, including the patient’s baseline characteristics, device sensitivity, acute event and post-event related probabilities, treatment effects, utility, and costs. The relevant costs were varied by ± 20%, whereas the risk and utility values were tested by their higher and lower quartiles. The 15 most impactful factors that yielded the widest differences were presented. A probabilistic sensitivity analysis (PSA) was also performed with 1,000 samples, and appropriate distributions were fitted to all model variables to capture uncertainty and variability in the estimates.

#### Scenario analysis

The scenario analysis examined the impact of a hypothetical ICM with up to a 30-percentage point lower sensitivity than that provided by LINQ ICM. The range is designed to reflect plausible variations, recognizing that unpublished data for some devices may lead to uncertainties about sensitivity range [63–65]. Furthermore, we performed a probability sensitivity analysis to account for uncertainty on the potential variations.

## Results

### Base case analysis

In the base case, when LINQ ICM was compared with the SoC, the findings revealed an additional 0.23 QALYs gained and an incremental cost of £1,980 over a patient’s lifetime. ICM versus SoC enabled the detection of an additional 0.254 AF cases and the avoidance of 0.071 secondary strokes, including 0.026 severe and fatal strokes, with a stroke-related cost savings of £924. The ICER for LINQ ICM versus the SoC was £8,670 per QALY. The detailed results of the base case analysis are listed in Table 5.

**Table 5:** Summary of base case results per patient

Results – Base Case	LINQ ICM	SoC	Difference: LINQ ICM versus SoC
AF detection	0.333	0.079	0.254
Total IS stroke	0.587	0.657	-0.071
* Severe and fatal IS stroke*	0.201	0.227	-0.026
QALYs	7.454	7.226	0.228
Total costs (£)	£26,609	£24,629	£1,980
* Total stroke event costs (£)*	£7,532	£8,456	£-924
ICER versus SoC (£)	-	-	£8,670

*AF* atrial fibrillation, *IS* ischaemic stroke, *QALYs* quality-adjusted life years, *ICER* incremental cost-effectiveness ratio

### Sensitivity analysis

**Fig. 2:**
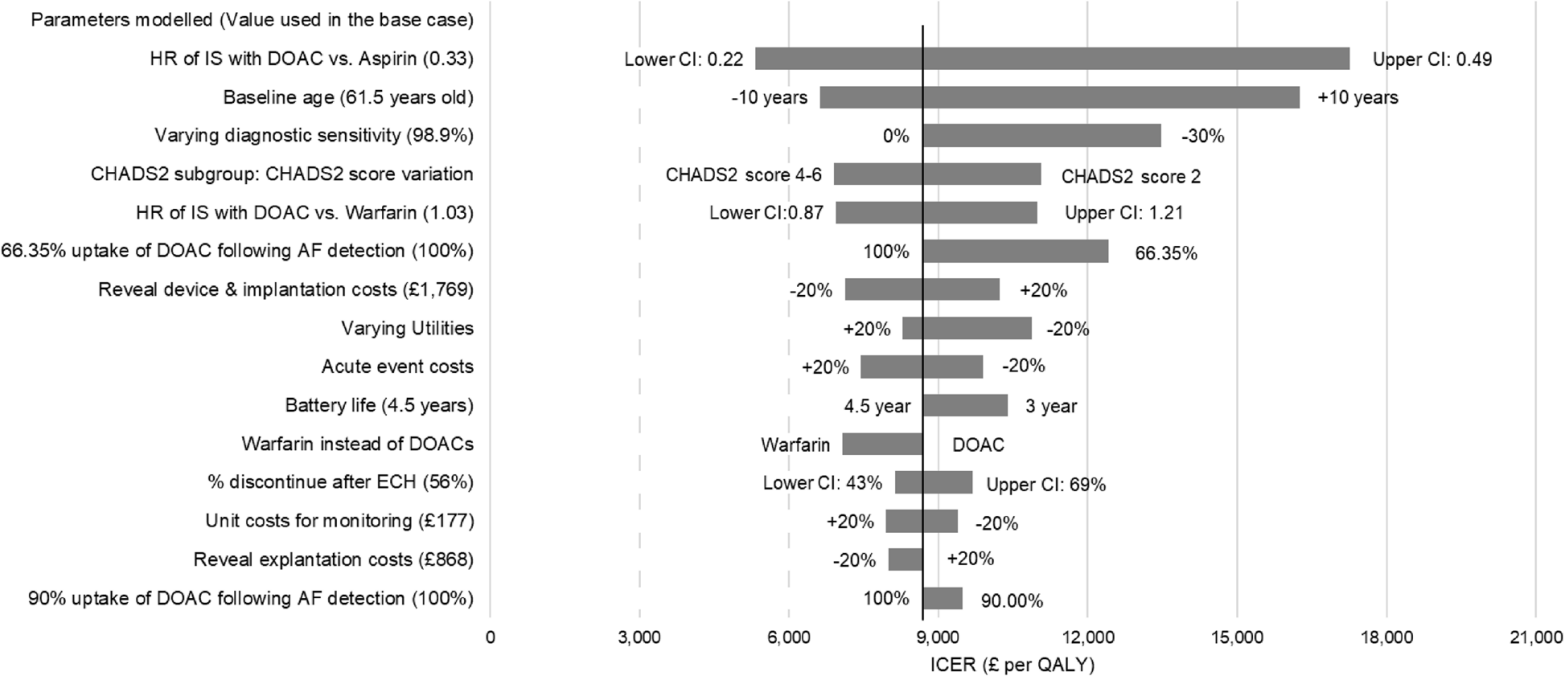
ICM versus SoC ICER: Tornado diagram

Figure 2 shows the 15 most important factors that OWSA evaluated out of the many parameters that were modelled. The three key drivers influencing cost-effectiveness of LINQ ICM versus SoC were the efficacy of DOACs versus aspirin, patients’ baseline age, and differences in diagnostic sensitivity. The least effective DOAC would result in an ICER of £17,258, whereas patients who are 10 years older would have an ICER of £16,256. A lower sensitivity ICM would raise the ICER to £13,479. The probabilistic sensitivity analysis presented in Table 6 shows the average 95% confidence interval of costs, QALYS and other outcomes., which is close to the base case result.

**Table 6:** Summary of probabilistic sensitivity analysis results per patient

Results: Probabilistic sensitivity analysis	LINQ ICM (95% CI)	SoC (95% CI)	Difference: LINQ ICM versus SoC
Total IS stroke	0.584 (0.436–0.733)	0.658 (0.511–0.812)	0.074
QALYs	7.464 (6.919–7.994)	7.229 (6.667–7.757)	0.234
Total costs (£)	£26,900 (£21,599–£33,061)	£24,907 (£20,239–£30,125)	£1,993
* Total stroke event costs (£)*	£7,558 (£5,281–£7,558)	£8,528 (£6,199–£8,528)	£-970
ICER versus SoC (£)	Reference	-	£8,502

*CI* confidence interval, *AF* atrial fibrillation, *IS* ischaemic stroke, *QALYs* quality-adjusted life years, *ICER* incremental cost-effectiveness ratio

### Scenario analysis

We examined the impact of varying ICM AF detection sensitivity in the scenario analysis, with the results presented for every 10% change in Table 7. For example, a comparison between LINQ ICM (98.9%) and a device with 30% points lower sensitivity (68.9%) indicated that a device with lower sensitivity would miss 0.095 AF detections, leading to 0.024 additional secondary strokes per patient. Thus, LINQ ICM resulted in 37.5% higher odds of detecting AF as well as 9.5% lower odds of ischaemic strokes (5.2% lower odds of severe or fatal ischaemic strokes). Moreover, using a device with less sensitivity led to a higher ICER versus SoC at £13,479 per QALY, a 55.5% increase on the ICER of LINQ ICM versus SoC.

**Table 7:** Summary of scenario analysis by varying diagnostic sensitivity

Results - Scenario Analyses	LINQ ICM	ICM with	vs.	ICM with	vs.	ICM with	vs.
Sensitivity	98.9%	88.9%	LINQ ICM	78.9%	LINQ ICM	68.9%	LINQ ICM
AF detection	0.333	0.302	*-0.031*	0.270	*-0.063*	0.238	*-0.095*
Total IS stroke per	0.587	0.595	*0.008*	0.603	*0.016*	0.611	*0.024*
* Severe and fatal IS stroke*	0.201	0.204	*0.003*	0.207	*0.006*	0.210	*0.009*
QALYs	7.454	7.429	*-0.026*	7.403	*-0.051*	7.377	*-0.078*
Total costs	£26,609	£26,627	*£18*	£ 26,644	*£35*	£26, 661	*£52*
* Total stroke event costs*	£7,532	£7,635	*£103*	£7,739	*£208*	£7, 845	*£314*
ICER versus SoC	£8,670	£9, 852	*£1*, *182*	£11, 392	*£2*,*721*	£13, 479	*£4*, *809*

*AF* atrial fibrillation, *IS* ischaemic stroke, *QALYs* quality-adjusted life years, *ICER* incremental cost-effectiveness ratio

**Fig. 3:**
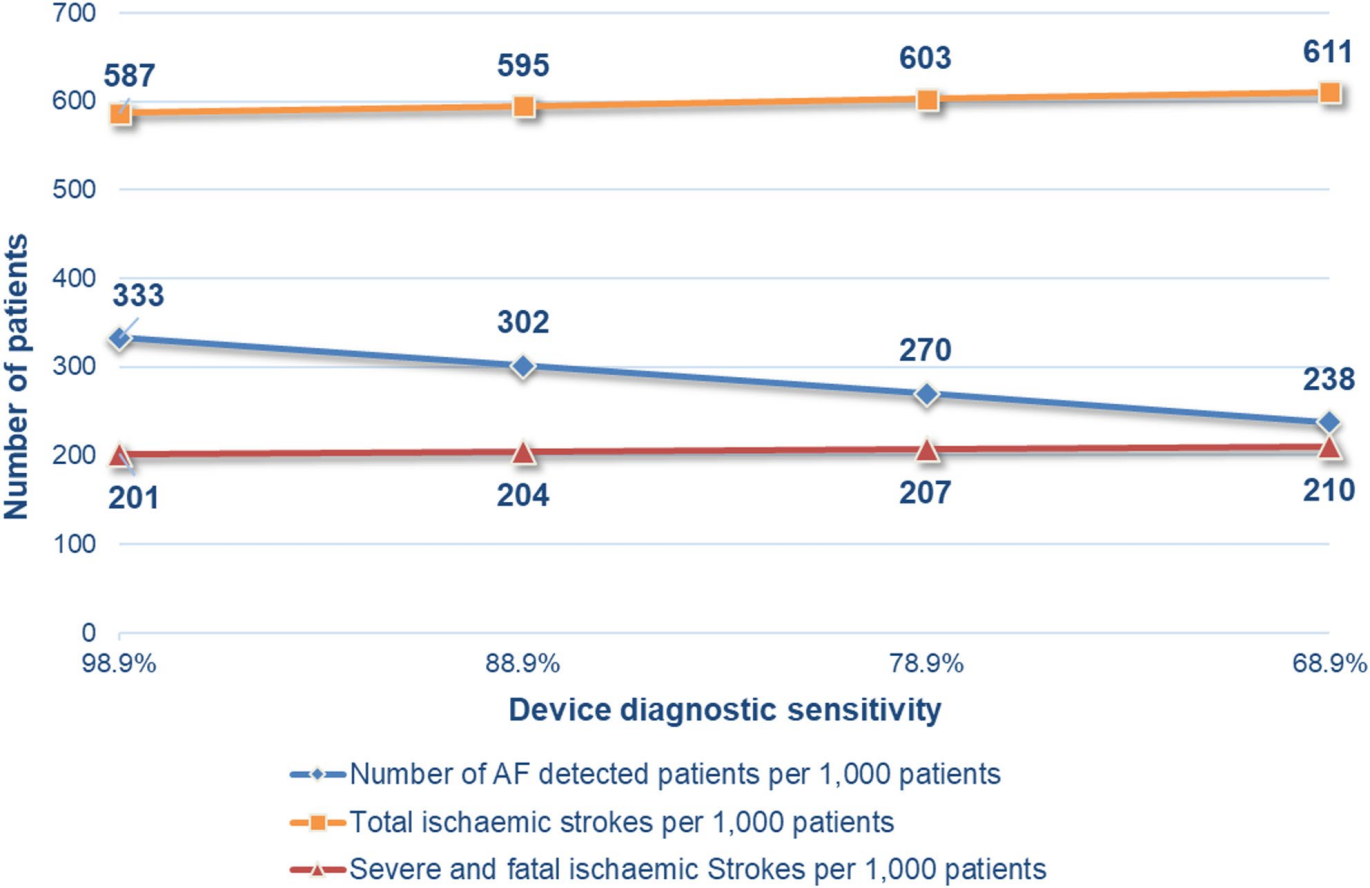
Number of AF detected and ischaemic strokes per 1,000 patients in the model

Figure 3 depicts the trend of AF detection, total ischaemic strokes, and severe and fatal ischaemic strokes, aggregated based on 1,000 patients, across various AF detection sensitivity levels. As the AF detection rate decreases, the incidence of ischaemic strokes, as well as severe and fatal ischaemic strokes, increases.

**Fig. 4:**
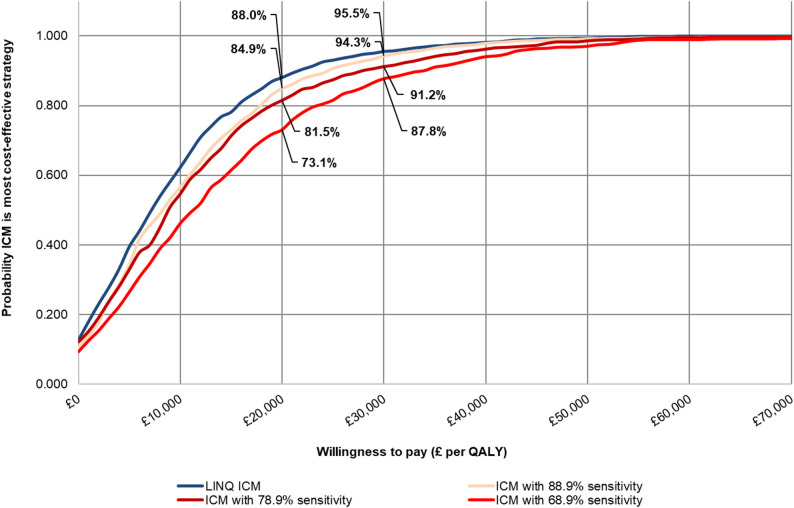
Cost-effectiveness acceptability curve: All ICMs versus SoC

The probabilistic sensitivity analysis of devices with varied sensitivity indicated that at a threshold of £20,000 per QALY, LINQ ICM had an 88.0% probability of being cost-effective versus SoC; this probability reached 95.5% at £30,000. In contrast, an ICM device that is 30% points lower in diagnostic sensitivity than LINQ ICM was 73.1% cost-effective at £20,000 and 87.8% at the £30,000 threshold. These results are displayed on the cost-effectiveness acceptability curve in Fig. 4.

**Fig. 5:**
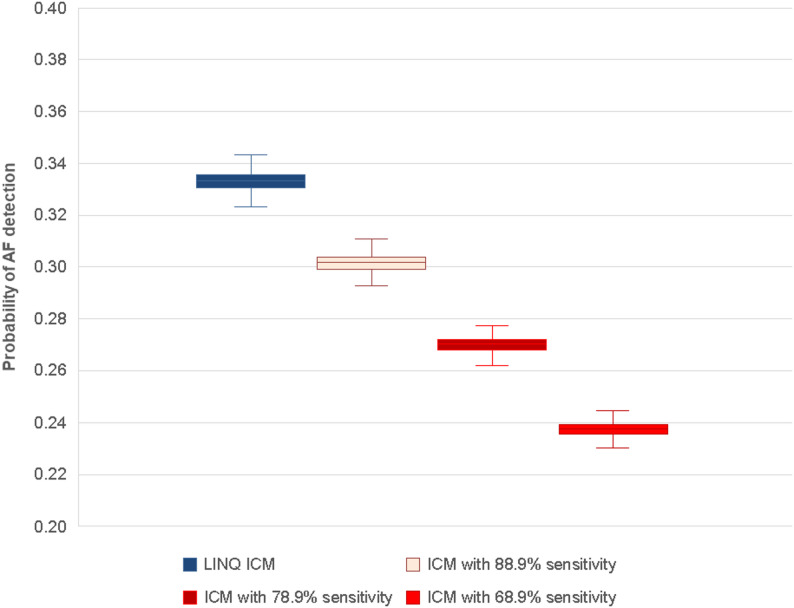
Box plot: Probability of AF detection in all ICMs

It is also evident from the box plot under PSA that AF detection significantly decreases when the device diagnostic sensitivity is lowered (Fig. 5). Looking at the median point, LINQ ICM (98.9%) reveals 0.333 AF detections in a patient’s lifetime, whereas the lowest sensitivity ICM (68.9%) detects only 0.238 AF cases per patient, resulting in more than a quarter of AF cases being missed.

## Discussion

### Summary of the main results

This study confirms that the LINQ ICM is more cost-effective than SoC for prolonged cardiac monitoring of CS patients. Despite the higher initial cost, the ICM leads to a longer life expectancy and better HRQoL. The additional life-years gained are due to increased AF detection, which subsequently prevents secondary ischaemic strokes by switching aspirin to DOACs upon AF detection. These results are in line with those of a previous cost-effectiveness study evaluating ICM versus SoC in the UK NHS setting [23, 52]. Compared with previous work where the ICER was found to be £17,175 [13], this study found a considerably lower ICER at £8,670 per QALY gained. This reduction is likely to be attributed to a 19% decrease in DOAC treatment costs, resulting in a lower economic burden to prevent secondary strokes; a 32% increase in stroke-related costs, making stroke prevention economically more appealing; and an extension of ICM battery life from 3 to 4.5 years, allowing for the detection of more AF events over time. This shows that the initial investment in the ICM accrues benefit over time by improving patient outcomes and lessening the overall burden on the healthcare system.

One-way sensitivity analysis revealed the top three key drivers influencing cost-effectiveness: the efficacy of DOACs versus aspirin, patients’ baseline age, and differences in diagnostic sensitivity. The first driver is a potential pharmacologically controllable factor depending on the DOAC chosen, whereas the second is an uncontrollable factor when treating patients. The third driver, the diagnostic sensitivity of the ICM, is also considered a controllable factor linked to the device chosen.

The literature shows secondary strokes to be more severe. Case-fatality after secondary stroke has been estimated to be 42% [32]. Jørgensen et al., 1997 found that the relative risk of death was almost doubled following recurrent vs. first ever stroke [31]. Recurrent strokes have also been shown to be very disabling. As an example, patients who survived a recurrent stroke experienced substantially more severe functional disability if they had a contralateral recurrence [31]. Experiencing a recurrent stroke lowered quality of life in the OXVASC study below the level experienced after a primary stroke [33].

### Scenario analysis – varied diagnostic sensitivity

To prevent secondary strokes and related costs, higher diagnostic sensitivity is essential for identifying AF and lowering the ICER per QALY. Holding everything else constant, varied diagnostic sensitivity has consequences on patients’ risk of ischaemic stroke owing to the difference in the AF detection rate, indicating a clinically meaningful impact. For example, in the scenarios explored, LINQ ICM compared with a device 30% less sensitive would enable 37.5% higher odds of AF detection and a subsequent 9.5% lower odds of ischaemic stroke. Compared with a device with 20% points lower sensitivity, LINQ ICM enables 35.0% higher odds of AF detection, leading to a reduction in strokes, or 6.4% lower odds of ischaemic stroke.

An ideal ICM should also be able to detect AF as soon as the first event occurs. A device with lower sensitivity, while potentially able to detect AF at a later stage, is likely to miss earlier events that therefore remain undetected and untreated. The later the AF is detected, the greater the risk of secondary stroke the patient bears, as well as the greater the economic burden the patient carries [66].

Understanding the impact of diagnostic sensitivity on patients’ health outcomes in the long run is paramount. As AI algorithms are developed to reduce the diagnostic burden or false alerts, the preservation of diagnostic sensitivity sometimes decreases as true events are filtered out [65, 67, 68]. In the longer term, it is likely that the diagnostic sensitivity of some market-available products will slowly decline over time if a balance between sensitivity and detection burden is not reached. In this case, devices with decreased sensitivity could fail to perform their core function: detecting all relevant arrhythmias.

### Strengths and limitations

This is the first study to model the clinical and economic impact of the ICM AF detection sensitivity from the perspective of the UK NHS. It also adds complexity to the previous NICE-appraised models by modelling the risk of a second recurrent stroke, strengthening the model’s validity [23, 52]. Additionally, the study makes use of the most recently published clinical costs for patients with stroke and bleeding [53]. It also assumes a 4.5-year battery life to estimate the most recently used LINQ ICM device [48]. Extensive sensitivity analyses were conducted to address uncertainty, testing multiple scenarios through OWSA and varying scores of parameters in the PSA.

Nevertheless, this analysis has several limitations. The sensitivity analysis revealed that the hazard ratios (HRs) for ischaemic stroke between DOACs and aspirin, as well as warfarin, were key drivers of uncertainty. In this study, direct or indirect HR comparisons were drawn from multiple existing published studies, as a comprehensive network meta-analysis encompassing all treatments is lacking [27–30]. Although reasonable time and effort were invested in synthesising the data used, the indirect comparison may have contributed to the uncertainty of the risk of stroke. Additionally, the HRs used in this analysis were assumed to remain constant over time. This was primarily due to the type of evidence available in the published literature and a lack of access to individual patient data from trials of aspirin and DOACs to confirm the validity of time-constant HRs. However, recent evidence has shown that risk factors such as age and post-stroke disability have a time-dependent effect on all-cause mortality risk in patients with ischaemic stroke during the first 7.5 years after stroke [69]. Furthermore, Alvarado-Bolanos et al., 2024 found that patients with known AF before a stroke or TIA and patients with newly-detected AF on a 12-lead ECG at the time of their stroke admission have a time-dependent risk of recurrent ischaemic stroke in the short term (12 months post-stroke) [70]. Taken together, these studies suggest that the relative risk of recurrent stroke and all-cause mortality might change over time and that time-constant HRs may not be valid over short to medium terms. Based on the authors’ assessment of the studies used in this analysis, the Kaplan-Meier curves for the risk of recurrent stroke do not show any crossing or indicators of convergence, thus supporting the use of time-constant HRs. However, to confirm this interpretation of the evidence, Schoenfeld residual plots and Pearson correlation of ranked times and residuals would need to be generated from individual patient data, which was not available to the authors. Given the short-term nature of the time dependency of stroke risk and the lifetime horizon of this analysis, using time-varying HRs may lead to more accurate cost-effectiveness estimates, but is unlikely to substantially change the results.

Specificity, defined as the rate of true negative ECGs seen with an ICM or SoC approach, and false positive rates were not included in this analysis because they were deemed to have a very limited impact on patient outcomes, and thus, cost-effectiveness. In particular, AF specificity performance for the Reveal LINQ ICM and conventional approaches is over 90%, meaning that a high degree of heart rhythm is correctly identified as normal sinus or non-AF rhythms. Additionally, for all patients with underlying AF and who are being monitored with ICMs or conventional approaches, physicians review all ECG data generated from AF alerts to determine whether they are true or false positives, the duration of the AF episodes as well as the frequency. Because physicians are able to filter out any false positive ECGs, the clinical care and management of patients with AF is unlikely to change as a result of false positives. Therefore, limited impact would be expected on treatment decisions and downstream risks of clinical outcomes and associated costs. However, specificity and false positive alerts do play a role in the workload of physicians and monitoring centres. While the impact of this workload burden is at least partially covered by the inclusion of monitoring costs for ICMs in this analysis; the addition of specificity in cost-effectiveness analyses may provide a more accurate picture of the economic impact of ICMs.

This model does not account for societal costs associated with ischaemic strokes. The relatively young cohort in this model suggests that many might still be employed. Overlooking societal costs means that the impact on the workforce and productivity was neglected. Excluding societal costs fails to account for the broader economic impact of stroke on productivity and long-term care needs. Despite an underestimation of the true burden of stroke, they were also not considered in the model as NICE and other HTA bodies do not include them in their assessments.

Finally, although some factors are known to affect the risk of strokes, there is no consensus on the length of AF episodes that represent increased risk [71]. In the CRYSTAL-AF study, the threshold for detecting AF episodes with ICM was set at longer than 2 min, whereas in the SoC arm, episodes longer than 30 s were included. However, a subgroup analysis of the CRYSTAL-AF study revealed that 95% of patients who were diagnosed with AF in the ICM arm experienced an episode longer than 6 min, suggesting that the results would be robust even if a more strict threshold was assumed [72].

### Implications for policy and future research

Decreased ICM diagnostic sensitivity leads to increases in the ICER such that the approach becomes much less cost-effective. To sustain cost-effectiveness, policymakers could prioritise ensuring that ICM devices with high diagnostic sensitivity are used.

In addition to diagnostic sensitivity, specificity is also clinically relevant in determining data review burden and has indirect implications for the costs of healthcare professionals [73]. However, it has not been included in previous cost-effectiveness analyses. Future studies should include specificity into the modelling to demonstrate the associated potential economic burden.

## Conclusion

This study demonstrates that the diagnostic sensitivity of ICMs plays a significant role in determining their clinical effect and cost-effectiveness of prolonged cardiac monitoring in patients with cryptogenic stroke. The LINQ ICM is highly cost-effective from the perspective of the UK NHS by reliably detecting AF episodes, enabling early initiation of DOAC therapy, which reduces the risk of recurrent ischaemic strokes and the associated healthcare costs. These findings reinforce the value of high-sensitivity ICMs, in improving patient outcomes while ensuring cost-effectiveness.

## Supplementary Information

Supplementary Material 1.

## Data Availability

All publicly available data analysed during the current study are identified either in the manuscript, supplementary information files or in cited source references. All other data are available upon reasonable request by contacting the corresponding author: [Jonas.villinger@medtronic.com].

## Abbreviations

AF: Atrial fibrillation
CHADS_2_: Congestive heart failure, hypertension, age ≥ 75, diabetes mellitus, stroke/transient ischemia attack/thromboembolism
CRNM: Clinically relevant non-major
CS: Cryptogenic Stroke
DOAC: Direct oral anticoagulant
ECG: Electrocardiogram
ECH: Extracranial Haemorrhage
GI: Gastrointestinal
HRQoL: Health-related quality of life
HS: Haemorrhagic stroke
ICH: Intracranial haemorrhage
ICM: Insertable cardiac monitor
ICERs: Incremental cost-effectiveness ratios
IS: Ischemic stroke
mRS: Modified rankin scale
NHS: National health service
OWSA: Deterministic one-way sensitivity analysis
PSA: Probabilistic sensitivity analysis
QALYs: Quality-adjusted life years
SoC: Standard of care
UK: United Kingdom
