# Supplementary material for: Economic and clinical impact of diagnostic sensitivity for the use of insertable cardiac monitors to detect atrial fibrillation in cryptogenic stroke patients in the United Kingdom
Source: BMC Cardiovasc Disord. 2026 Apr 24;26:486. doi: 10.1186/s12872-026-05871-0 (PMC13251038; doi:10.1186/s12872-026-05871-0)
Supplement: Supplementary file 1 — Supplementary Material 1. [file 12872_2026_5871_MOESM1_ESM.docx]

**Supplementary tables**

**Table 1 | Baseline characteristics from CRYSTAL-AF trial**

| **CHADS_2_ score** | **CHADS_2_ 2** | **CHADS_2_ 3** | **CHADS_2_ 4** | **CHADS_2_ 5** | **CHADS_2_ 6** | **All patients** |
| --- | --- | --- | --- | --- | --- | --- |
| **N** | 150 | 183 | 84 | 23 | 1 | 441 |
| **Weighted** | 34% | 41% | 19% | 5% | 0% | 100% |
| **Mean age** | 54.3 | 62.1 | 68.6 | 77.9 | 78.0 | 61.5 |
| **% male** | 61.3% | 68.9% | 64.3% | 34.8% | 0.0% | 63.5% |

**Table 2 | Per-cycle risks by AF status and treatment**

| **CHADS_2_ score** | **Aspirin [with AF]** | **Aspirin [no AF]** | **DOAC [with AF]** | **Warfarin [with AF]** |
| --- | --- | --- | --- | --- |
|  | *Risk of ischaemic stroke (first recurrent)* | | | |
| CHADS_2_ 0 | 0.002 | 0.001 | 0.001 | 0.001 |
| CHADS_2_ 1 | 0.006 | 0.004 | 0.002 | 0.002 |
| CHADS_2_ 2 | 0.011 | 0.008 | 0.005 | 0.004 |
| CHADS_2_ 3 | 0.022 | 0.015 | 0.009 | 0.009 |
| CHADS_2_ 4 | 0.028 | 0.019 | 0.011 | 0.011 |
| CHADS_2_ 5 | 0.032 | 0.021 | 0.013 | 0.013 |
| CHADS_2_ 6 | 0.036 | 0.024 | 0.014 | 0.014 |
| Weighted average | 0.020 | 0.013 | 0.008 | 0.008 |

AF: atrial fibrillation; DOAC: direct anticoagulant

**Table 3 | Distribution of ischaemic stroke severity on the basis of various treatments**

| **Ischaemic stroke severity** | **APX** | **DBG 110mg** | **DBG 150mg** | **RVX** | **ASA** | **WARF** | **Overall Average** |
| --- | --- | --- | --- | --- | --- | --- | --- |
| % mild (mRS 0-2) | 53.0% | 35.0% | 35.0% | 49.0% | 36.0% | 45.0% | **42.2%** |
| % moderate (mRS 3-4) | 21.0% | 28.0% | 22.0% | 18.0% | 38.0% | 30.0% | **26.2%** |
| % severe (mRS 5) | 8.0% | 10.0% | 8.0% | 6.0% | 15.0% | 10.0% | **9.5%** |
| % fatal (mRS 6) | 18.0% | 27.0% | 35.0% | 27.0% | 11.0% | 15.0% | **22.2%** |
| Total | 100.0% | 100.0% | 100.0% | 100.0% | 100.0% | 100.0% |  |
| Source | [1] | [1] | [1] | [1] | [1] | [2] |  |

mRS: modified Rankin Scale; APX: apixaban; DBG: dabigatran; RVX: rivaroxaban; ASA: aspirin; WARF: warfarin

**Table 4 | Distribution of IS severity on the basis of first or second recurrent stroke**

| **Stroke severity** | **First recurrent stroke** | **Second recurrent stroke after moderate IS*** | **Second recurrent stroke after severe IS** |
| --- | --- | --- | --- |
| Mild | 42.2% | - | - |
| Moderate | 26.2% | 45.2% | - |
| Severe | 9.5% | 16.4% | 30.0% |
| Fatal | 22.2% | 38.3% | 70.0% |

IS: ischaemic stroke

**Table 5 | ICH- and ECH-related bleeding probabilities**

|  | **APX** | **DBG 110mg** | **DBG 150mg** | **RVX** | **ASA** | **WARF** | **Overall Average** | |
| --- | --- | --- | --- | --- | --- | --- | --- | --- |
| *ICH* | | | | | | | | |
| % ICH are HS | 77.0% | 64.0% | 41.0% | 57.0% | 55.0% | 64.0% | **59.7%** | |
| % HS are mild | 23.0% | 35.0% | 35.0% | 49.0% | 7.0% | 20.0% | **28.2%** | |
| % HS are moderate | 32.0% | 28.0% | 22.0% | 18.0% | 20.0% | 15.0% | **22.5%** | |
| % HS are severe | 10.0% | 10.0% | 8.0% | 6.0% | 27.0% | 12.0% | **12.2%** | |
| % HS are fatal | 35.0% | 27.0% | 35.0% | 27.0% | 46.0% | 53.0% | **37.2%** | |
| *ECH* | | | | | | | | |
| % ECH are GI bleed | 38.0% | 41.0% | 49.0% | 45.0% | 39.0% | 39.0% | **41.8%** | |
| Source | [1] | | | | | [2] | |  |

ICH: intracranial haemorrhage; HS: haemorrhagic stroke; ECH: extracranial haemorrhage; GI: gastrointestinal; APX: apixaban; DBG: dabigatran; RVX: rivaroxaban; ASA: aspirin; WARF: warfarin

**Table 6 | Treatment effects for bleeding events on the basis of different sources**

| **Variable** | **Mean** | **Standard Error** | **Source** |
| --- | --- | --- | --- |
| **ICH** |  | | |
| HR apixaban vs aspirin | 0.80 | 0.71 | RR from[3], Table 5 |
| HR apixaban vs warfarin | 0.37 | 0.12 | RR from[3], Table 5 |
| HR aspirin vs warfarin | 0.46 | 0.46 | Indirect comparison from[3], Table 5 |
| Peto OR DOAC vs warfarin | 0.47 | 0.07 | RR from[3], Table 5 |
| **GI bleed** |  | | |
| HR apixaban vs aspirin | 0.80 | 0.72 | [4] |
| HR apixaban vs warfarin | 0.83 | 0.28 | Indirect comparison [4-6] |
| HR aspirin vs warfarin | 1.04 | 1.12 | [1, 2]; Average across all drugs based on all randomised patients |
| Peto OR DOAC vs warfarin | 1.22 | 0.18 | [6] |
| **CRNM bleed** |  | | |
| HR aspirin vs DOAC | 0.87 | 0.13 | Reciprocal value of [7] |
| HR DOAC vs warfarin | 0.85 | 0.02 | [2, 8-10]; Average across treatment effects in all randomised patients |

ICH: intra-cranial haemorrhage; HR: hazard ratio; OR: odds ratio; RR: relative risk; DOAC: direct anticoagulant

**Table 7 | Annual risk of bleeding events on the basis of various treatments**

|  | **Aspirin** | **Warfarin** | **DOAC** |
| --- | --- | --- | --- |
| All ICH | 0.0055 | 0.0120 | 0.0056 |
| GI bleed | 0.0115 | 0.0111 | 0.0135 |
| CRNM bleed | 0.0786 | 0.1066 | 0.0904 |

ICH: intracranial haemorrhage; GI: gastrointestinal; DOAC: direct anticoagulant; CRNM: clinical relevant non-major

**Table 8 | Estimated excess mortality on the basis of severity of recurrent stroke events**

| **Risk factor - Adjusted values for model** | **Hazard ratio** | **Notes/Source** |
| --- | --- | --- |
| Mild stroke | 2.56 | [11] |
| Moderate stroke | 4.63 | [11] |
| Severe stroke | 13.19 | [11] |

**Table 9 | Utility values in various health states**

| **Health state/event** | **Mean utility in study** | **Source** |
| --- | --- | --- |
| Health state utility |  |  |
| CRYSTAL-AF baseline | 0.774 | [12]; data on file |
| Mild stroke event (IS or HS) | 0.730 | [13], Table 2 |
| Moderate stroke event (IS or HS) | 0.500 | [13], Table 2 |
| Severe stroke event (IS or HS) | 0.130 | [13], Table 2 |
| Post-mild stroke (IS or HS) | 0.727 | [13], mean of 60-month data (Table 2); NIHSS ≤ 3 |
| Post-moderate stroke (IS or HS) | 0.582 | [13], mean of 60-month data (Table 2); NIHSS ≤ 3 |
| Post-severe stroke (IS or HS) | 0.397 | [13], mean of 60-month data (Table 2); NIHSS > 10 |
| Other ICH event | 0.700 | [13], 1-month data (Table 2) for subarachnoid haemorrhage |
| Utility gains |  |  |
| History of AF | -0.014 | [13], average of 1 and 60-month data (Table 4) |
| Recurrent stroke event | -0.150 | [13], 1 month data (Table 4) |
| Post recurrent stroke | -0.068 | [13], 60-month data (Table 4) |
| CRNM bleed* | -0.058 | [2, 14] |
| ECH† | -0.151 | [2, 14] |

Notes: * CRNM bleed disutility assumed to last for 2 days; † ECH disutility assumed to last for 2 weeks

IS: ischaemic stroke; HS: haemorrhagic stroke; ICH: intracranial haemorrhage; AF: atrial fibrillation; CRNM: clinical relevant non-major; ECH: extracranial haemorrhage

**Table 10 | Health state utility multipliers**

| **Health state** | **φ** |
| --- | --- |
| No AF | 0.9406 |
| AF | 0.9227 |
| Post-mild stroke - No AF | 0.8733 |
| Post-mild stroke - AF | 0.8566 |
| Post-moderate stroke - No AF | 0.6991 |
| Post-moderate stroke - AF | 0.6858 |
| Post-severe stroke - No AF | 0.4769 |
| Post-severe stroke - AF | 0.4678 |
| **Acute event** |  |
| Mild recurrent stroke | 0.7705 |
| Moderate recurrent stroke | 0.5278 |
| Severe recurrent stroke | 0.1372 |
| Other ICH | 0.9270 |
| ECH | 0.9942 |
| CRNM bleed | 0.9997 |

AF: atrial fibrillation; ICH: intracranial haemorrhage; ECH: extracranial haemorrhage; CRNM: clinical relevant non-major

**Table 11 | Tests performed per person per year in the control arm of CRYSTAL-AF**

| **Period** | **No test** | **ECG** | **Holter 24H** | **Holter 48H** | **Holter 7D** |
| --- | --- | --- | --- | --- | --- |
| 0-12 months | 0.3066 | 0.549289 | 0.0630 | 0.022512 | 0.0585 |
| 12-24 months | 0.5080 | 0.397931 | 0.0362 | 0.007235 | 0.0506 |
| 24-36 months | 0.5818 | 0.313663 | 0.0209 | 0 | 0.0836 |

ECG: electrocardiogram

**Table 11 | Summary of base case results breakdown of events and costs**

| **Results – Base Case** | **LINQ ICM** | **SoC** | **Difference: LINQ ICM versus SoC** |
| --- | --- | --- | --- |
| ***Events*** |  |  |  |
| **Ischaemic stroke** | 0.5867 | 0.6574 | -0.0707 |
| Mild | 0.2257 | 0.2509 | -0.0252 |
| Moderate | 0.1597 | 0.1792 | -0.0195 |
| Severe | 0.0609 | 0.0686 | -0.0077 |
| Fatal | 0.1405 | 0.1587 | -0.0182 |
| **Haemorrhagic stroke** | 0.0451 | 0.0423 | 0.0028 |
| Mild | 0.0116 | 0.0107 | 0.0008 |
| Moderate | 0.0109 | 0.0103 | 0.0006 |
| Severe | 0.0058 | 0.0055 | 0.0003 |
| Fatal | 0.0168 | 0.0158 | 0.0011 |
| **Other ICH** | 0.0305 | 0.0286 | 0.0019 |
| **ECH** | 0.3976 | 0.3617 | 0.0358 |
| **CRNM Bleed** | 1.1045 | 1.0101 | 0.0944 |
| ***Costs*** |  |  |  |
| **Diagnostic costs** | £3,063 | £1,042 | £1,980 |
| **Health state costs** | £12,376 | £11,786 | £2,022 |
| **Event related costs** | £11,170 | £11,801 | £590 |
| Total stroke event costs | £7,532 | £8,456 | -£631 |
| Total bleed event costs | £3,638 | £3,345 | -£924 |

**Supplementary File References**

1. Lip GYH *et al.*: **Cost-effectiveness of apixaban versus other new oral anticoagulants for stroke prevention in atrial fibrillation**. *Clinical therapeutics* 2014, **36**(2):192-210.e120.

2. Dorian P *et al.*: **Cost-effectiveness of apixaban vs. current standard of care for stroke prevention in patients with atrial fibrillation**. *European heart journal* 2014, **35**(28):1897-1906.

3. Tawfik A *et al.*: **Systematic review and network meta-analysis of stroke prevention treatments in patients with atrial fibrillation**. *Clinical pharmacology : advances and applications* 2016, **8**:93-107.

4. Diener H-C *et al.*: **Apixaban versus aspirin in patients with atrial fibrillation and previous stroke or transient ischaemic attack: a predefined subgroup analysis from AVERROES, a randomised trial**. *The Lancet Neurology* 2012, **11**(3):225-231.

5. Easton JD *et al.*: **Apixaban compared with warfarin in patients with atrial fibrillation and previous stroke or transient ischaemic attack: a subgroup analysis of the ARISTOTLE trial**. *The Lancet Neurology* 2012, **11**(6):503-511.

6. Ntaios G *et al.*: **Nonvitamin-K-antagonist oral anticoagulants in patients with atrial fibrillation and previous stroke or transient ischemic attack: a systematic review and meta-analysis of randomized controlled trials**. *Stroke* 2012, **43**(12):3298-3304.

7. Connolly SJ *et al.*: **Apixaban in Patients with Atrial Fibrillation**. *New England Journal of Medicine* 2011, **364**(9):806-817.

8. Connolly SJ *et al.*: **Dabigatran versus Warfarin in Patients with Atrial Fibrillation**. *New England Journal of Medicine* 2009, **361**(12):1139-1151.

9. Giugliano RP *et al.*: **Edoxaban versus Warfarin in Patients with Atrial Fibrillation**. *New England Journal of Medicine* 2013, **369**(22):2093-2104.

10. Patel MR *et al.*: **Rivaroxaban versus Warfarin in Nonvalvular Atrial Fibrillation**. *New England Journal of Medicine* 2011, **365**(10):883-891.

11. Huybrechts KF *et al.*: **The prognostic value of the modified Rankin Scale score for long-term survival after first-ever stroke. Results from the Athens Stroke Registry**. *Cerebrovascular diseases (Basel, Switzerland)* 2008, **26**(4):381-387.

12. Sanna T *et al.*: **Cryptogenic Stroke and Underlying Atrial Fibrillation**. *New England Journal of Medicine* 2014, **370**(26):2478-2486.

13. Luengo-Fernandez R *et al.*: **Quality of life after TIA and stroke: ten-year results of the Oxford Vascular Study**. *Neurology* 2013, **81**(18):1588-1595.

14. Sullivan PW *et al.*: **Catalogue of EQ-5D Scores for the United Kingdom**. *Medical Decision Making* 2011, **31**(6):800-804.
